# Genome-wide association analysis reveals genetic loci and candidate genes for feeding behavior and eating efficiency in Duroc boars

**DOI:** 10.1371/journal.pone.0183244

**Published:** 2017-08-16

**Authors:** Rongrong Ding, Jianping Quan, Ming Yang, Xingwang Wang, Enqin Zheng, Huaqiang Yang, Disheng Fu, Yang Yang, Linxue Yang, Zicong Li, Dewu Liu, Gengyuan Cai, Zhenfang Wu, Jie Yang

**Affiliations:** 1 College of Animal Science and National Engineering Research Center for Breeding Swine Industry, South China Agricultural University, Guangdong, P.R. China; 2 National Engineering Research Center for Breeding Swine Industry, Guangdong Wens Foodstuffs Group Co., Ltd, Guangdong, P.R. China; Universita degli Studi di Bologna, ITALY

## Abstract

Efficient use of feed resources is a challenge in the pork industry because the largest variability in expenditure is attributed to the cost of fodder. Efficiency of feeding is directly related to feeding behavior. In order to identify genomic regions controlling feeding behavior and eating efficiency traits, 338 Duroc boars were used in this study. The Illumina Porcine SNP60K BeadChip was used for genotyping. Data pertaining to individual daily feed intake (DFI), total daily time spent in feeder (TPD), number of daily visits to feeder (NVD), average duration of each visit (TPV), mean feed intake per visit (FPV), mean feed intake rate (FR), and feed conversion ratio (FCR) were collected for these pigs. Despite the limited sample size, the genome-wide association study was acceptable to detect candidate regions association with feeding behavior and eating efficiency traits in pigs. We detected three genome-wide (*P* < 1.40E-06) and 11 suggestive (*P* < 2.79E-05) single nucleotide polymorphism (SNP)-trait associations. Six SNPs were located in genomic regions where quantitative trait loci (QTLs) have previously been reported for feeding behavior and eating efficiency traits in pigs. Five candidate genes (*SERPINA3*, *MYC*, *LEF1*, *PITX2*, and *MAP3K14*) with biochemical and physiological roles that were relevant to feeding behavior and eating efficiency were discovered proximal to significant or suggestive markers. Gene ontology analysis indicated that most of the candidate genes were involved in the development of the hypothalamus (GO:0021854, *P* < 0.0398). Our results provide new insights into the genetic basis of feeding behavior and eating efficiency in pigs. Furthermore, some significant SNPs identified in this study could be incorporated into artificial selection programs for Duroc-related pigs to select for increased feeding efficiency.

## Introduction

Pork is a major meat resource for humans, providing over ~37% of all meat in 2012–2014[1]. The increasing demand for pork has prompted breeders around the world to significantly improve swine production. However, the cost of feed is the single largest variable for swine production, ranging from 50% to 85% of the total production cost[2]. The cost of feed can be further compounded by competition between animal agriculture, human food, and biofuel industries resulting in augmentation of the demand for grain and higher grain prices[3]. Improving feed conversion ratio (FCR) and other feeding behavioral traits is important to solve these problems.

With the development of computerized systems that record feed intake and related measurements, extensive research investigations on feeding behavior and eating efficiency have been performed[1, 4, 5]. Several studies have shown a strong correlation between the feeding behavioral traits and eating efficiency traits in livestock. For instance, Do DN *et al*. showed that the average daily feed intake (DFI) is positively correlated to FCR, and total time spent at feeder per day (TPD) is negatively correlated to mean feed intake rate (FR) [6]. Rauw *et al*. reported that faster-consuming pigs have higher levels of food intake, improved growth rates, and accumulated more body fat[7]. However, simple correlation studies between the feeding behavioral traits and eating efficiency traits could not provide a direct approach insight into the genetic determinants of control of FCR and other feeding behavioral traits.

In previous decades, quantitative trait loci (QTL) mapping was the method of choice for scientists to understand the genetics of complex traits such as FCR. With the development of genetic linkage studies, thousands of QTLs have been detected for economically important traits in livestock[8]. For example, 55 QTLs for DFI and 179 QTLs for FCR have been identified in different pig chromosomes (http://www.animalgenome.org/cgi-bin/QTLdb/SS/index). However, the biggest challenge for QTL mapping is to locate a large interval of at least 20 centimorgans (cM) in length. With the development of dense genomic markers and the significant reduction in sequencing costs, genome-wide association studies (GWAS) have proven to be a useful and powerful method in addressing this challenge in human[9] and other animal species[10, 11].

Duroc is widely used in pig production based on its excellent growth traits. It is significant for pig practitioners and even human feeding behavior researchers to identify potential genes that are associated with feeding behavior and eating efficiency. Therefore, understanding the genetic determinants controlling FCR and other feeding behavioral traits based on GWAS is crucial to improving Duroc breeding programs and enhancing their feeding efficiency. In pigs, there is an increasing number of association studies on Duroc purebred to detect SNPs associated with polygenetic traits, such as obesity-related traits[12]. Here, we conducted GWAS for feeding behavior and eating efficiency traits to identify the precise locations of QTLs for such important traits in Duroc pigs.

## Materials and methods

### Ethics statement

The experimental procedures used in this study met the guidelines of the Animal Care and Use Committee of the South China Agricultural University (SCAU) (Guangzhou, People’s Republic of China). The Animal Care and Use Committee of the SCAU (Approval number SCAU#0017) approved all the animal experiments described in this study.

### Animals and phenotype recording

Between 2011 and 2014, a total of 338 Duroc boars (average IBS value was 0.733 ± 0.015) were collected from the Guangdong Wen’s Foodstuffs Group Co., Ltd. (Guangdong, China). All 338 boars were approximately fed under uniform feeding conditions for measurements of feed behaviors and eating efficiency traits during the fattening period (approximately 11 weeks) from 30 kg to 100 kg live weight. These pigs were group housed in half-open cement-floor pens (10 animals in each pen, with an average of 2 m^2^ per pig). Each animal was labeled a unique electric tag on its ear during the testing period. In the present study, both 338 boars had feeding behavior phenotypic data, and 324 had FCR phenotypic data. Both phenotypic data were collected by using the Osborne FIRE Pig Performance Testing System (Kansas, American) of Guangdong Wen’s Foodstuffs Group Co., Ltd. (Guangdong, China)[1]. The time, duration, feed consumption, and weight of each individual were recorded at every visit. Average daily feed intake (DFI) was calculated based on the total amount of recorded total feed intake divided by the number of corresponding feed days. The following feeding behavior and eating efficiency traits were defined and calculated for each boar: average DFI (kg/d), TPD (min), NVD, TPV (= TPD/NVD), FPV (kg), FR (= DFI/TPD, g/min), and FCR[4, 6].

### Genotyping and quality control

Genomic DNA was extracted from ear tissues using the traditional method of phenol-chloroform and adjusted to a concentration of 50 ng/μL[13]. DNA quality was assessed by ratios of light absorption (A_260/280_ and A_260/230_) and electrophoresis. Genotyping was performed using the Porcine SNP60 Beadchip of Illumina (San Diego, CA, USA)[14], which contains 61,565 SNP markers across the entire genome. A total of 338 samples were genotyped. QC was conducted using Plink v1.07[15]. Briefly, animals with call rates of > 0.95 (MIND) and SNPs with call rates of > 0.99 (GENO), minor allele frequency > 0.01 (MAF), *P* value>10^−6^ for the HWE test were included. Moreover, all of the SNPs located on the sex chromosome and unmapped regions were excluded from the analysis. A final set of 35,791 informative SNPs from 338 pigs were used for the subsequent analyses.

### GWAS

Genome-wide association analysis is a research method that is generally used to determine the correlation between high-density genetic markers and complex traits. The R package GenABEL was used to perform genome-wide association analysis under a general linear mixed model[16, 17]. The model included a random polygenic effect for which the variance-covariance matrix was proportionate to genome-wide identity-by-state[18]. The following mixed model was used to perform GWAS: *Y~μ+Xb+Kw+Sc+Za+e*, where *Y* is the vector of phenotypes; *μ* is the overall mean; *b* is the vector of fixed effects including pigsty and year-season effects; *w* is the vector of live weight of individuals when the measurement is completed considered as covariate; *c* is the vector of SNP effects; *a* is the vector of random additive genetic effects with *a*~N(0, Gσ_α_^2^), where *G* is the genomic relationship matrix calculated from the 60K SNP makers in the Duroc population and σ_α_^2^ is the polygenetic additive variance; *K* is the regression coefficient of live weight of individuals when the measurement is completed; and *e* is the vector of residual errors with *e*~N(0, Iσ_e_^2^), where *I* is the identity matrix and σ_e_^2^ is the residual variance. *X*, *S*, and *Z* are incidence matrices for *b*, *c*, and *a* respectively.

The Bonferroni method was used to determine the genome-wide significance threshold, in which the conventional *P*-value was divided by the number of tests performed[19]. According to the Bonferroni method, the genome-wide significant (significant) and chromosome-wide significant (suggestive) thresholds were *P* < 0.05/N and *P* < 1/N, respectively, where N is the number of SNPs tested in the analyses. In the present study, the significant and suggestive thresholds were 1.40E-06 (0.05/35791) and 2.79E-05 (1/35791), respectively.

### Quantile-quantile plots

Because population stratification greatly impacts GWAS reliability, quantile-quantile (Q–Q) plot analysis is considered an effective way to determine the reliability of the GWAS results. In a Q–Q plot, the horizontal axis represents the expected -log_10_*P* value, and the vertical axis represents the observed -log_10_*P* value. The diagonal line represents y = x, the shaded region shows a 95% confidence interval based on a Beta distribution. An overall deviation above the diagonal identity line is generally suggestive of severe population stratification[20]. Deviations from the diagonal line indicate that either the assumed distribution is incorrect or that the sample contains values arising in some other manner, similar to that generated by true association[21]. The Q–Q plot was constructed using the R software.

### Haplotype block analysis

The software Plink v1.07 [http://pngu.mgh.harvard.edu/purcell/plink/] and Haploview [http://www.broadinstitute.org/scientific-community/science/programs/medical-and-population-genetics/haploview/haploview] were utilized for haplotype block analysis. Linkage disequilibrium blocks were defined using Haploview 4.2 based on SNPs with MAF values of > 0.05, Mendel errors of < 2, and *P*-value in the HWE test of < 10^−3^. We performed haplotype block analysis, which requires at least two suggestive SNPs in a chromosome[1, 22].

### Gene ontology analysis

SNP positions from the *Sus scrofa* 10.2 genome version were downloaded from www.animalgenome.org/pig/. The Ensembl annotation of the *Sus scrofa* 10.2 genome version was employed to find genes that were nearest the significant SNPs [http://ensembl.org/Sus_scrofa/Info/Index]. To annotate significant SNP positions to previously mapped QTLs in pigs, all QTL data in pigs were downloaded from http://www.animalgenome.org/cgi-bin/QTLdb/SS/download?file=gbpSS_10.2 (accessed on April 3, 2016)[23]. Gene Ontology analysis was performed on the GO website [http://geneontology.org/][24].

## Results

### Phenotypes and quality control (QC) of genotypes

A summary of the statistics of the seven traits are presented in Table 1. Prior to GWAS analysis, we assessed the distribution of all phenotypes by using the Shapiro test[25]. All phenotypic data conformed to the Gaussian distribution. After QC-filtering of the genotypic data, 18,792 markers that showed low (< 1%) minor allele frequencies, 489 markers with low (< 99%) call rate, and 840 markers not within Hardy-Weinberg equilibrium (HWE) (*P* < 10^−6^) were excluded from the analysis. A total of 6,002 SNPs located on the sex chromosome and unknown chromosomal regions were thus removed. A final set of 35,791 SNPs and 338 pigs was retained for subsequent GWAS analysis. The number of markers on each chromosome and average distances between two markers after QC are presented in S1 Table. The average physical distance between two neighboring SNPs on the same chromosome was approximately 66.8 kb and ranged from 54.7 (SSC14) to 85.8 kb (SSC1).

**Table 1:** Descriptive statistical analysis of feeding behavior and eating efficiency in a male Duroc population.

Trait^1^	Units	N	Mean	SD	Min	Max
DFI	kg	338	1.985	0.278	1.463	2.617
TPD	min	338	65.469	16.478	37.596	128.807
NVD	count	338	7.805	2.963	3.096	17.06
TPV	min	338	9.54	3.798	4.077	20.618
FPV	kg	338	0.295	0.117	0.107	0.712
FR	g/min	338	31.77	7.521	14.042	48.111
FCR	kg/kg	324	2.06	0.244	1.7	2.7

^1^DFI: Total daily feed intake, FPV: Mean feed intake per visit, FR: Mean feed intake rate, NVD: Number of visits to the feeder per day, TPD: Total time spent at feeder per day, TPV: Time spent to eat per visit, FCR: Feed conversion ratio.

Mean, standard deviation (SD), minimum (min) and maximum (max) values are presented for all of the phenotypes included in the association study (N).

### Significant SNPs and haplotype block analysis

**Fig 1:**
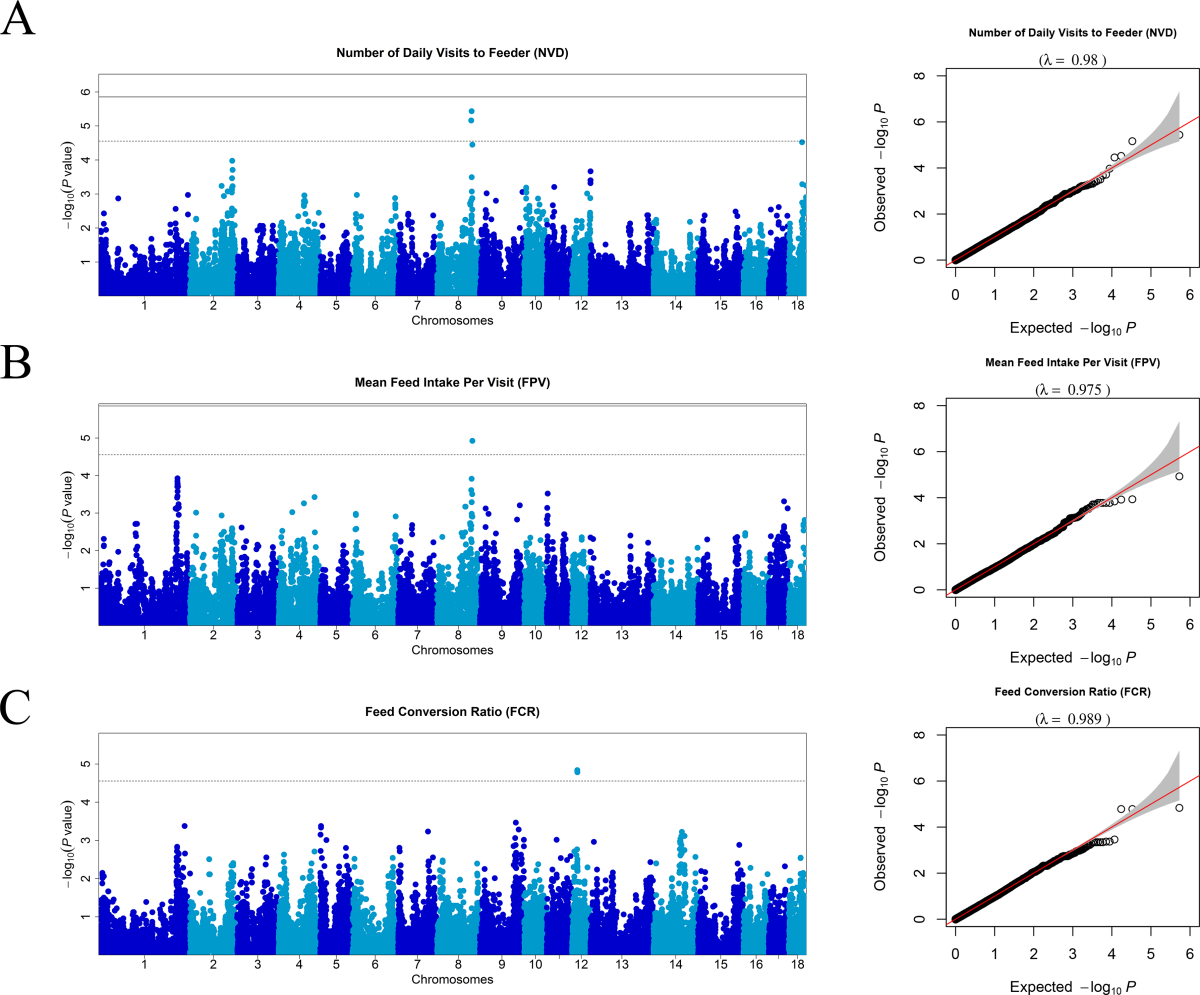
Manhattan plots of genome-wide association studies for eating efficiency and feeding behavior in male Duroc pigs. The inserted quantile–quantile (Q–Q) plots in the right show the observed versus expected log p-values. In the Manhattan plots, negative log10 *P* values of the quantified SNPs were plotted against their genomic positions. Different colors indicate various chromosomes. The solid and dashed lines indicate the 5% genome-wide and chromosome-wide Bonferroni-corrected thresholds, respectively. On the vertical axis, Manhattan plot and Q-Q plot for the number of visits to the feeder per day (NVD), FPV, and feed conversion ratio (FCR), respectively.

**Fig 2:**
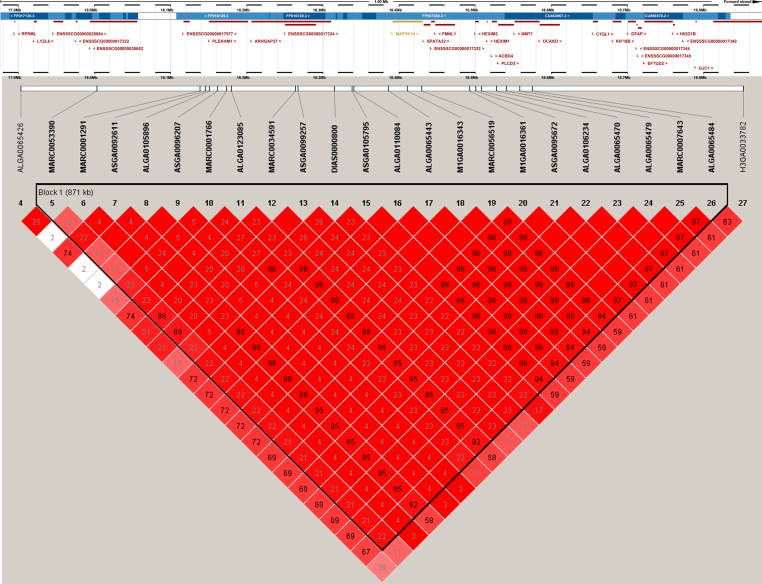
The linkage disequilibrium (LD) block and candidate genes in the significantly associated region on SSC12. LD blocks are marked with triangles. Values in boxes are LD (r^2^) between SNP pairs and the boxes are colored according to the standard Haploview color scheme: LOD >2 and D’ = 1, red; LOD >2 and D’<1, shades of pink/red; LOD <2 and D’ = 1, blue; LOD <2 and D’<1, white (LOD is the log of the likelihood odds ratio, a measure of confidence in the value of D’). Annotated genes in the chromosomal region retrieved from the Ensemble genome browser (www.ensembl.org/Sus_scrofa/Info/Index).

Manhattan plots of GWAS of all traits after QC and the Q–Q plots are shown in Fig 1 and S1 Fig. The average genomic inflation factors (λ) of the GWAS for all feeding behavior and feed conversion ratio traits were 0.991, ranging from 0.973 (average duration of each visit, TPV) to 1.03 (DFI), which suggests that there was little or no evidence of residual population structure effects on test statistic inflation[26]. The tag (significant and suggestive) SNPs detected by the associated test for the seven traits are shown in Table 2. In total, three significant and 11 suggestive SNPs were identified. The three significant SNPs were associated with DFI, whereas no suggestive SNP was associated with FR and TPD. The number of suggestive SNPs associated with DFI, FCR, NVD, TPV, and mean feed intake per visit (FPV) was 4, 3, 2, 1, and 1, respectively. The chromosomes and exact positions based on *Sus scrofa* Genebuild 10.2 as well as genes neighboring the tag SNPs are listed in Table 2. Two of 14 SNPs were located within intronic regions of known genes, and one haplotype block was detected in genomic regions affecting FCR on SSC12 (Fig 2). Approximately 22 SNPs of the haplotype block were located in SSC12 and ranged in size from 17.9 Mb to 18.8 Mb, three of these SNPs significantly affected FCR. Furthermore, 25 genes were identified within the haplotype block (Fig 2).

**Table 2:** Tag SNPs and closest genes for feeding behavior and eating efficiency traits.

Trait^1^	SNP ID	SSC^2^	Location (bp)^3^	Adjusted P value^4^	Nearest gene	Gene location^5^	Distance/bp^6^	*Homo sapiens* homologs
DFI	ASGA0036538	7	123,104,663	6.10E-08	*SERPINA3*	7:123,094,888–123,101,407	-3256	*SERPINA3*
	DRGA0006936	6	132,729,420	3.90E-07	*LRRC7*	6:132,544,632–132,612,363	-117057	*LRRC7*
	DRGA0016772	17	55,848,150	9.00E-07	*ENSSSCG00000007456*	17:55,754,008–55,846,536	-1614	-
	ASGA0025032	5	21,273,976	1.80E-06	*ENSSSCG00000021160*	5:21,279,720–21,280,679	+5744	-
	ALGA0037698	6	152,852,810	4.00E-06	*PIK3R3*	6:152,798,324–152,914,703	Within	*PIK3R3*
	ASGA0018273	4	11,639,918	1.60E-05	*ENSSSCG00000005961*	4:11,122,206–11,124,073	-515845	-
	ASGA0018324	4	12,174,119	2.44E-05	MYC	4:12,778,444–12,783,481	604325	MYC
FPV	ALGA0049273	8	122,208,906	1.20E-05	LEF1	8:122,085,975–122,204,345	-4561	LEF1
NVD	ASGA0087256	8	119,705,102	3.65E-06	PITX2	8:119,920,838–119,926,111	+215736	PITX2
	MARC0008579	8	118,421,256	6.87E-06	*AP1AR*	8:118,369,021–118,401,177	-20079	*AP1AR*
TPV	ALGA0008796	1	268,986,440	1.92E-05	*ENSSSCG00000029421*	1:268,964,060–268,973,843	-22380	-
FCR	MARC0053390	12	17,941,131	1.44E-05	*LRRC37B*	12:17,946,401–17,959,278	+5270	*LRRC37B*
	MARC0034591	12	18,338,026	1.64E-05	*MAP3K14*	12:18,391,607–18,431,381	+53581	*MAP3K14*
	ASGA0099257	12	18,342,922	1.64E-05	*MAP3K14*	12:18,391,607–18,431,381	Within	*MAP3K14*

^1^DFI: Total daily feed intake, FPV: Mean feed intake per visit, NVD: Number of visits to the feeder per day, TPV: Time spent to eat per visit, FCR: Feed conversion ratio.

^2^*Sus scrofa* chromosome

^3^SNP positions in Ensembl.

^4^Genome-wide significant associations are underlined.

^5^Gene location in Ensembl.

^6^+/-: The SNP located in the upstream/downstream of the nearest gene; NA: not assigned.

### Comparison with previously mapped QTL in pigs

A total of four SNPs associated with DFI were identified within the genomic region where QTLs for feed intake traits and/or feed conversion ratio were previously been mapped in pigs (Table 3). One SNP on SSC1 associated with TPV and one locus on SSC6 that was associated with DFI was located on previously reported QTL regions for average daily gain in different pig populations. Moreover, eight SNPs were located in the genomic region where QTLs were previously detected by GWAS or linkage maps for backfat and obesity-related traits in pigs.

**Table 3:** Comparative mapping of tag SNPs with previous QTLs reported in the pig QTL database (as of April 3, 2016) and previous GWAS results.

Traits^1^	SNP	SSC^2^	SNP position (bp)^3^	Starting QTL position (bp)^4^	Ending QTL position (bp)^5^	QTL_ID^6^	Corresponded trait in the QTL database
DFI	ASGA0036538	7	123,104,663	121,429,704	124,338,264	65,101	Fat weight (total)
	DRGA0006936	6	132,729,420	94,382,869	146,365,886	2,879	Daily feed intake
	DRGA0016772	17	55,848,150	45,880,990	67,855,516	16,842	Backfat on the last rib
	ASGA0025032	5	21,273,976	3,688,083	34,660,429	979	Feed intake
	ALGA0037698	6	152,852,810	149,326,918	153,593,360	19,382	Average daily gain
	ASGA0018273	4	11,639,918	7,237,639	12,618,993	5,162	Feed conversion ratio
	ASGA0018324	4	12,174,119	7,237,639	12,618,993	5,162	Feed conversion ratio
FPV	ALGA0049273	8	122,208,906	108,610,930	139,007,531	17,782	Backfat on the last rib
NVD	ASGA0087256	8	119,705,102	108,610,930	139,007,531	17,782	Backfat on last rib
	MARC0008579	8	118,421,256	108,610,930	139,007,531	17,782	Backfat on last rib
TPV	ALGA0008796	1	268,986,440	87,993,461	279,498,596	22,269	Average daily gain
FCR	MARC0053390	12	17,941,131	17,226,344	23,672,762	3,967	Backfat between the last 3rd and 4th lumbar
	MARC0034591	12	18,338,026	17,226,344	23,672,762	3,967	Backfat between the last 3rd and 4th lumbar
	ASGA0099257	12	18,342,922	17,226,344	23,672,762	3,967	Backfat between the last 3rd and 4th lumbar

^1^DFI: Total daily feed intake, FPV: Mean feed intake per visit, NVD: Number of visits to the feeder per day, TPV: Time spent to eat per visit, FCR: Feed conversion ratio.

^2^*Sus scrofa* chromosome

^3^SNP positions in Ensembl

^4^Starting position of the mapped QTL in the QTL database

^5^Ending position of the mapped QTL in the QTL database

^6^Identity of QTL in the pig QTL database or published literature

### Candidate genes at significant or suggestive level

The aim of the present study was to identify and characterize genes that were related to novel feeding behavior and eating efficiency. To obtain more credible results, we attempted to reduce the number of potential genes based on biochemical and physiological roles that were relevant to feeding behavior and eating efficiency traits. Ultimately, a total of five candidate genes were obtained. Of these, serpin family A member 3 (*SERPINA3*), myc proto-oncogene protein (*MYC*) were correlated to DFI; lymphoid enhancer binding factor 1 (*LEF1*), which is a potential functional candidate gene, was associated with FPV; paired-like homeodomain 2 (*PITX2*), a potential functional candidate gene, was associated with NVD; and mitogen-activated protein kinase kinase kinase 14 (*MAP3K14*) was correlated with FCR. We then conducted gene ontology analysis of the five candidate genes, which indicated that most of the genes were involved in the development of the hypothalamus (GO:0021854, *P*< 0.0398).

## Discussion

### Comparison of QTLs identified in this study with those in previous studies

GWAS is a powerful tool for the genetic analysis of important traits of domestic animals. Recently, some authors have reported the results of GWAS for feeding behavior and eating efficiency in pig. For example, Do DN *et al*. identified 16 significant SNPs and 76 suggestive SNPs that were associated with feeding behavior traits in the Duroc population[4]. Of these SNPs, 36 SNPs were located in genome regions where QTLs for feeding behavior and/or feed intake traits were previously been reported in pigs[4]. In their study, a moderate threshold criterion was employed, in which loci with *P* < 5E-05 were considered as moderately genome-wide significant and those with *P* < 5E-04 were considered to be suggestively genome-wide significant. They finally found a large number of SNPs associated with behavior and/or feed intake traits, but this method also raised the possibility to detect false positive results. In our study, the Bonferroni correction, which is a stringent correction method, was employed to reduce the occurrence of false positive results. Compared to the results of previous studies, we detected a markedly lower number of tag SNPs; however, we believe that these SNPs provide promising markers to improve feed efficiency related traits in the Duroc population that we tested. Moreover, Guo *et al*. also identified six QTLs that were associated with eating efficiency and feeding behavior at suggestive and significance levels, and two QTLs were associated with more than one trait in a White Duroc × Erhualian F_2_ resource population[27]. Although progress has been made in identifying QTLs by GWAS, the majority of associations detected from GWAS are still for common variants. QTL caused by low-frequency or rare variants have not been efficiently identified in previous pig GWAS, especially in those using small sample size.

The present study detected a SNP (ALGA0008796) associated with TPV for average daily gain in Yorkshire pigs in SSC1[28]. Two SNPs (ASGA0018273 and ASGA0018324) associated with DFI for feed conversion ratio were identified in SSC4 of Large White×·Landrace × Leicoma pigs[29], and a SNP (ASGA0025032) associated with DFI for feed intake was localized on SSC5 of Meishan × Pietrain pigs[30]. Two SNPs (DRGA0006936 and ALGA0037698) associated with DFI were also identified in SSC6, one was situated in a region linked to DFI in Pietrain pigs[31], whereas the other was in a region related to average daily gain[32]. Some other QTLs and SNPs associated with feeding behavior were not found in regions that were previously reported to be related to feeding behavior, nevertheless, we compared our GWAS results for backfat and obesity-related traits with those of previous studies. We found that three SNPs (MARC0053390, MARC0034591 and ASGA0099257) associated with FCR on SSC12 were in previously mapped QTLs that spanned 40.2–64.7 (cM) for backfat between the last 3rd and 4th lumbar in a commercial four-way cross[33]. Moreover, two SNPs (ASGA0087256 and MARC0008579) associated with NVD and a SNP (ALGA0049273) associated with FPV on SSC8 were detected in regions for backfat on the last rib in an Iberian × Meishan F_2_ sow family[34]. The close proximity of the three SNPs suggests that the same gene also affects the NVD and FPV traits of backfat on the last rib. In general, six SNPs were located in genome regions where QTLs for feeding behavior and eating efficiency were previously reported in pigs.

Comparative mapping may facilitate in the validation of our results, as well as refine QTL regions and target candidate genes for complex traits such as feeding behavior. In addition, by comparing our results with those of previous QTL studies, we have determined that a large proportion of feeding behavior and eating efficiency traits located that have been previously reported. These observations are suggestive of a genetic correlation between feeding behavior and eating efficiency traits, as well as the occurrence of regional pleiotropic effects on feeding traits.

Furthermore, compared to conventional breeding, the marker-assisted selection (MAS) can speed up the breeding programs[35]. The MAS has been applied to improve reproduction rate, feed intake, growth rate and meat quality in commercial lines of pigs[36]. In this study, significant associations between polymorphisms and feeding behavior traits were revealed in the Duroc sire population. These associated variants provide novel molecular markers for the MAS to facilitate the genetic improvement of feeding behavior and eating efficiency traits in the Duroc sire population.

### Potential candidate genes

#### Potential candidate genes for average daily feed intake

Average daily feed intake is an important feeding behavior trait, and therefore is a major ethological concern of animal nutrition workers. The most significant locus, ASGA0036538, was closest to the *SERPINA3* gene (Table 2). The protein encoded by this gene is a plasma protease inhibitor and a member of the serine protease inhibitor class. Yang *et al*. reported that *SERPINA3* promotes endometrial cancer cell growth by regulating G2/M cell cycle checkpoints and apoptosis in human[37]. Graff *et al*. also identified a SNP within the *SERPINA3* could increase body weight significantly in human[38]. Moreover, Grubbs *et al*. reported that *SERPINA3* may play direct and important biological roles in the pathways that control residual feed intake (RFI) in young pigs[39]. Therefore, *SERPINA3* on SSC7 could be a candidate gene for DFI. The *MYC* gene was situated proximal to the significant SNP ASGA0018324, which is associated with DFI (Table 2). Palomero *et al*. determined that the *MYC* gene is involved in a feed forward loop transcriptional network that promotes leukemic cell growth[40]. Additionally, Malynn *et al*. found that *MYC* gene knockout mice tend to show lower weight and slower growth rate[41]. Therefore, the *MYC* gene on SSC4 could be a candidate gene for DFI.

The marker ALGA0037698 on SSC6, which is located on fourth intron of the *PIK3R3* gene, was associated with DFI (Table 2). Previous studies have shown that the gene *PIK3R3* is involved in cancer development, such as gastric cancer[42], ewing Sarcoma[43] and metastatic colorectal cancer[44]. However, no report to date has described a link between DFI and *PIK3R3* in any species. In addition, there were three genes (*ENSSSCG00000005961*, *ENSSSCG00000021160* and *ENSSSCG00000007456*) near the significant SNPs for DFI with unknown function. These three genes have not been studied to date and thus its function in pig has not been established. Moreover, the pig genome has not been completely annotated; therefore, additional research studies to better elucidate the association between these genes and DFI is warranted.

#### Potential candidate genes for FPV to the feeder

A single SNP with suggestive thresholds was associated with FPV. The lymphoid enhancer binding factor 1 (*LEF1*) was localized proximal to the SNP ALGA0049273 (Table 2). The protein encoded by this gene can bind to a functionally important site in the T-cell receptor-alpha enhancer, thereby conferring maximal enhancer activity. The biological functions of this gene include positive regulation of cell growth and cell proliferation in humans[45] and mice[46]. We thus inferred that the *LEF1* gene increases the production of energy based on body requirements by changing its feeding behavior. Therefore, *LEF1* on SSC8 could be a candidate gene for FPV.

#### Potential candidate genes for the number of visits to the feeder per day

The adaptor-related protein complex 1-associated regulatory protein (*AP1AR*) was located proximal to the suggestive SNP ASGA0087256, which is associated with NVD (Table 2). This protein is essential for adaptor protein complex 1 (AP-1)-dependent transport between the trans-Golgi network and endosomes. Diseases associated with *AP1AR* include gliosarcoma. No functional characterization of the gene in pigs has been conducted to date. The *PITX2* gene has been localized proximal to the suggestive SNP ASGA0087256, which is associated with NVD (Table 2). The encoded protein acts as a transcription factor and regulates the expression of procollagen lysyl hydroxylase, thereby influencing terminal differentiation of somatotrophs. Kappeler *et al*. found that regulating somatotroph function can change the food intake behavior of rats[47]. Senescence is related to a dysfunction of the somatotroph axis. Veyrat-Durebex *et al*. observed that aging Lou rats exhibit a decreased capacity to adjust feeding behavior to metabolic demands[48]. We inferred that the *PITX2* gene on SSC8 participates in regulating somatotroph function and could be thus a candidate gene for NVD.

#### Potential candidate genes for feed conversion ratio

The present study observed that the region on SSC12 showed the strongest association with FCR. The most significant SNP MARC0053390 association with FCR was located in an 871-kb LD block that comprised 25 genes (Fig 2). The leucine-rich repeat containing a 37B gene (*LRRC37B*) was located proximal to the top SNP, and this gene was related to human height[49]. The remaining two suggestive SNPs were also located within the 871-kb LD block, and one of these was closest to the *MAP3K14* gene, whereas the other marker, MARC0059507, was located on the *MAP3K14* gene and was associated with FCR (Table 2). This gene encodes mitogen-activated protein kinase kinase kinase 14, which is a serine/threonine protein-kinase. In mice, D-serine suppresses the intake of high-preference food[50]. Moreover, *MAP3K14* gene knockout mice showed a reduction in the weight of the mammary fat pad. In human, the *MAP3K14* gene is associated with multiple sclerosis[51], which involves motor and sensory dysfunction. Therefore, *MAP3K14* on SSC12 could be a candidate gene for FCR. This is the first report that associates the LD block on SSC12 with FCR.

#### Potential candidate genes for other feeding traits and gene ontology analysis

The *ENSSSCG00000029421* gene was located proximal to suggestive SNP ALGA0008796, which is associated with TPV. *ENSSSCG00000029421* is a *Sus scrofa* gene, and no homologs have been identified in *Homo sapiens*. To the best of our knowledge, no studies on this gene have been conducted to date. Based on TPD and FR, no SNP has reached the suggestive threshold, and thus these two traits were not further investigated in the present study.

Gene ontology analysis of the five candidate genes indicated that most of the genes were involved in the development of the hypothalamus. Bouret previously reported that the hypothalamus apparently plays an essential role in controlling appetite[52]. Moreover, Yoshimatsu *et al*. showed that hypothalamic histamine neurons play an important role in the central regulation of feeding behavior in rats, which is mainly controlled by leptin[53]. Likewise, Peng *et al*. showed that the *BMPR1A* gene regulates the development of hypothalamic circuits that are critical to the feeding behavior of mice[54]. These results suggest that the hypothalamus serves as the linkage between the five candidate genes and feeding behavior and eating efficiency traits.

One limitation of the current study is the considerable number of false negative genetic associations. Increasing the size of the study population may potentially prevent the generation of false-positive results. Fine-mapping and the identification of causal variants should also be based on the premise that we increase the sample size. All of these studies may serve as a foundation for better understanding the genetic mechanism underlying feeding behavior and eating efficiency.

## Conclusions

The present study provides a list of SNPs that are associated with feeding behavior and eating efficiency traits in pigs, and also offers valuable information on the genetic architecture and candidate genes for these traits. Fourteen significant or suggestive SNPs were detected in the Duroc sire population. Of these, two SNPs for NVD and one SNP for FPV were determined to be in close proximity on SSC8, thereby indicating a pleiotropic effect. Five candidate genes based on biochemical and physiological roles that were relevant to feeding behavior and eating efficiency were discovered closest to the significant or suggestive markers. Gene ontology analysis indicated that most of the genes were involved in hypothalamus development. The identification of several genomic regions and putative positional genes that were associated with feeding behavior and eating efficiency in the present study may contribute to marker-assisted selection in pig breeding.

## Supporting information

S1 Fig Manhattan plots of genome-wide association studies for eating efficiency and feeding behaviors in male Duroc pigs. The inserted quantile–quantile (Q–Q) plots in the right show the observed versus expected log p-values. In the Manhattan plots, negative log10 *P* values of the quantified SNPs were plotted against their genomic positions. Different colors indicate various chromosomes. The solid and dashed lines indicate the 5% genome-wide and chromosome-wide Bonferroni-corrected thresholds, respectively. On the vertical axis, Manhattan plot and Q-Q plot for total daily feed intake (DFI), total daily time spent at feeder per day (TPD), Time spent to eat per visit (TPV) and mean feed intake rate (FR), respectively.
(TIF)

S1 Table Distributions of SNPs after quality control and the average distance between adjacent SNPs on each chromosome. (DOCX)
