# Supplementary figures and images for: Genome-wide association analysis reveals genetic loci and candidate genes for feeding behavior and eating efficiency in Duroc boars
Source: PLoS One. 2017 Aug 16;12(8):e0183244. doi: 10.1371/journal.pone.0183244 (PMC5559094; doi:10.1371/journal.pone.0183244)

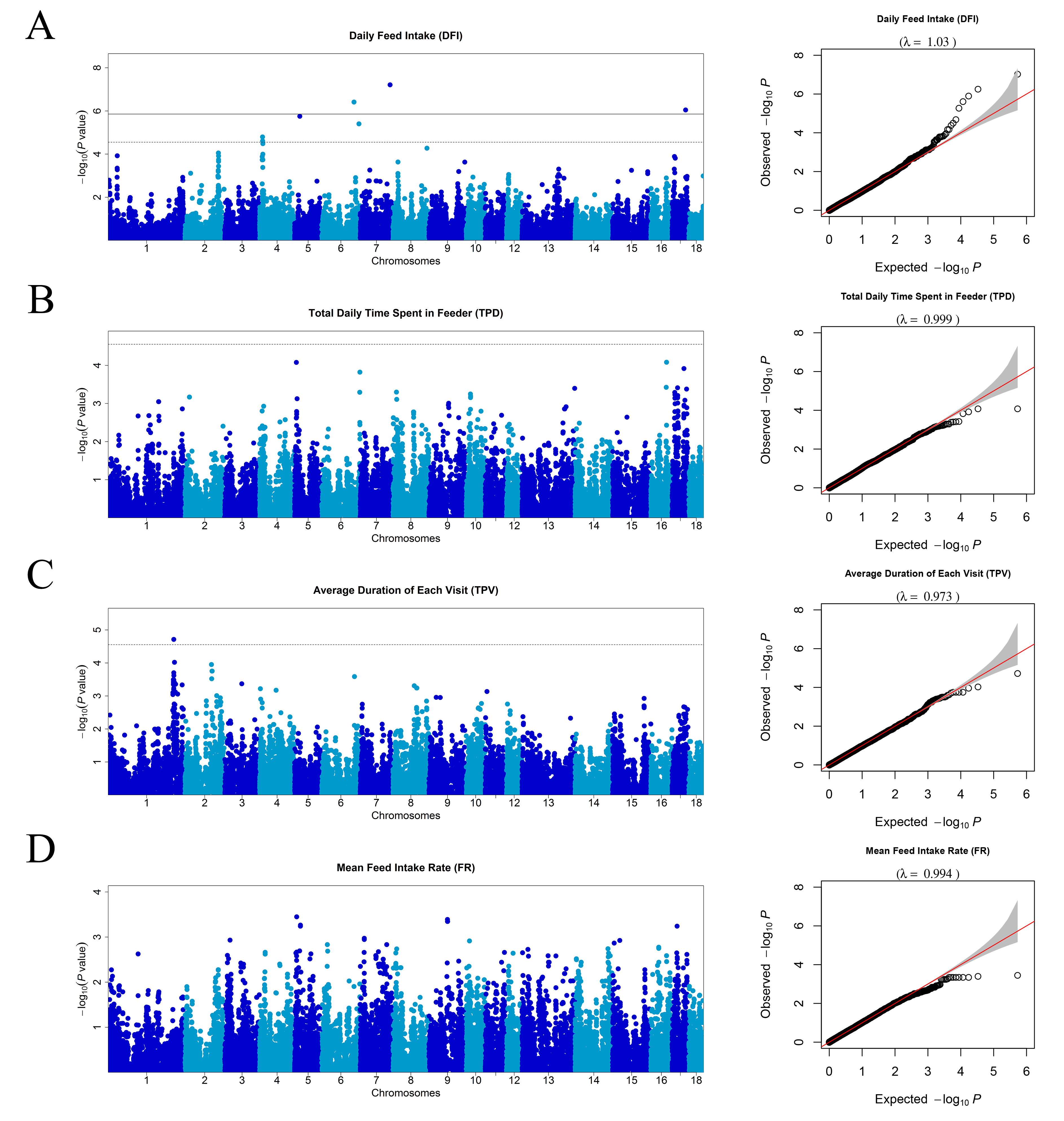

Supplement: S1 Fig — The inserted quantile–quantile (Q–Q) plots in the right show the observed versus expected log p-values. In the Manhattan plots, negative log10 P values of the quantified SNPs were plotted against their genomic positions. Different colors indicate various chromosomes. The solid and dashed lines indicate the 5% genome-wide and chromosome-wide Bonferroni-corrected thresholds, respectively. On the vertical axis, Manhattan plot and Q-Q plot for total daily feed intake (DFI), total daily time spent at feeder per day (TPD), Time spent to eat per visit (TPV) and mean feed intake rate (FR), respectively. (TIF) [file pone.0183244.s001.tif]
