# Supplementary material for: Genome-wide association analysis reveals genetic loci and candidate genes for feeding behavior and eating efficiency in Duroc boars
Source: PLoS One. 2017 Aug 16;12(8):e0183244. doi: 10.1371/journal.pone.0183244 (PMC5559094; doi:10.1371/journal.pone.0183244)
Supplement: S1 Table — (DOCX) [file pone.0183244.s002.docx]

**Supplementary Material**

**Genome-wide association analysis reveals genetic loci and candidate genes for** **feeding behavior and eating efficiency in Duroc boars**

Rongrong Ding^1$^, Jianping Quan^1$^, Ming Yang^2^, Xingwang Wang^1^, Enqin Zheng^1^, Huaqiang Yang ^1^, Disheng Fu^1^, Yang Yang^1^, Linxue Yang^1^, Zicong Li ^1^, Dewu Liu^1^, Gengyuan Cai^1,2^, Zhenfang Wu^1,2,*^, Jie Yang^1,*^

^1^College of Animal Science and National Engineering Research Center for Breeding Swine Industry, South China Agricultural University, Guangdong, P.R. China.

^2^National Engineering Research Center for Breeding Swine Industry, Guangdong Wens Foodstuffs Group Co., Ltd, Guangdong, P.R. China.

^$^These authors contributed equally to this work.

^*^Correspondence and requests for materials should be addressed to J.Y. (email: jieyang2012@hotmail.com) or Z.W. (email: wzfeamil@163.com)

**S1 Table. Distributions of SNPs after quality control and the average distance between adjacent SNPs on each chromosome.**

| **SSC** | **SNP no.** | **Physical size (Mb)^1^** | | **Mb/SNP** |
| --- | --- | --- | --- | --- |
| **1** | 3670 | 314.93 | 0.0858 | |
| **2** | 2447 | 162.28 | 0.0663 | |
| **3** | 1872 | 143.99 | 0.0769 | |
| **4** | 2499 | 143.40 | 0.0574 | |
| **5** | 1524 | 111.23 | 0.0730 | |
| **6** | 2047 | 157.53 | 0.0770 | |
| **7** | 2268 | 134.51 | 0.0593 | |
| **8** | 2159 | 147.27 | 0.0682 | |
| **9** | 2144 | 153.43 | 0.0716 | |
| **10** | 1361 | 77.73 | 0.0571 | |
| **11** | 1448 | 87.63 | 0.0605 | |
| **12** | 1141 | 63.41 | 0.0556 | |
| **13** | 2751 | 218.12 | 0.0793 | |
| **14** | 2806 | 153.53 | 0.0547 | |
| **15** | 2100 | 157.40 | 0.0750 | |
| **16** | 1370 | 86.69 | 0.0633 | |
| **17** | 1259 | 69.32 | 0.0551 | |
| **18** | 925 | 60.91 | 0.0658 | |
| **Total** | 35791 | 2443.31 |  | |

SNP, single nucleotide polymorphisms; SSC, *Sus scrofa* chromosome

^1^The physical size is based on *Sus scrofa* Build 9 (http://www.ensembl.org/Sus_scrofa/Info/Index)
